# Postinterventional surveillance at a dedicated valve unit is safe and reduces intensive care utilization after TAVR

**DOI:** 10.1007/s00392-025-02676-7

**Published:** 2025-06-02

**Authors:** B. Gonska, M. Krohn-Grimberghe, H. Kirindi, T. Stephan, J. Mörike, C. Buck, W. Rottbauer, D. Buckert

**Affiliations:** Department of Cardiology, Ulm University Heart Center, University of Ulm, Albert-Einstein-Allee 23, 89081 Ulm, Germany

**Keywords:** TAVR, TAVI, Valve unit, Postprocedural care, Intensive care unit

## Abstract

**Background:**

The postprocedural care pathway after transcatheter aortic valve replacement (TAVR) mostly includes monitoring patients for 24–48 h at an intensive care unit (ICU) or intermediate care unit (routine intensive care monitoring = ICM). To reduce the need for postprocedural intensive care surveillance, our center established a dedicated monitoring unit (valve unit = VU) for pre- and postprocedural care of TAVR patients.

**Methods:**

The aim of this prospective case–control study was to evaluate outcomes of patients directly before and after the introduction of the VU. Starting in April 2020 TAVR patients were directly transferred to a VU after the procedure with 24-h telemetric electrocardiogram (ECG) and non-invasive blood pressure monitoring, which was spatially integrated into a general cardiology ward. Patients with hemodynamic or respiratory instability, stroke, delirium, and severe bleeding complications were still directly transferred to the ICU.

**Results:**

796 consecutive patients treated with TAVR at our center were included. 592 patients had been treated during ICM and 204 after the establishment of the VU. The overall rate of events was similar before and after the implementation of the valve unit. 182 of 592 ICM patients developed study-specific endpoints (30.7%) compared to 60 of 204 VU patients (29.4%) (*P* value for difference: 0.87). VU patients showed a trend towards a lower rate of delirium (ICM 3.5% vs VU 1%, *p*-value 0.06).

**Conclusion:**

Introduction of a VU for patient monitoring after TAVR with prespecified criteria for postinterventional ICU surveillance reduced the percentage of postinterventional ICU admissions by 73% without increasing the overall rate of adverse events.

**Graphical Abstract:**

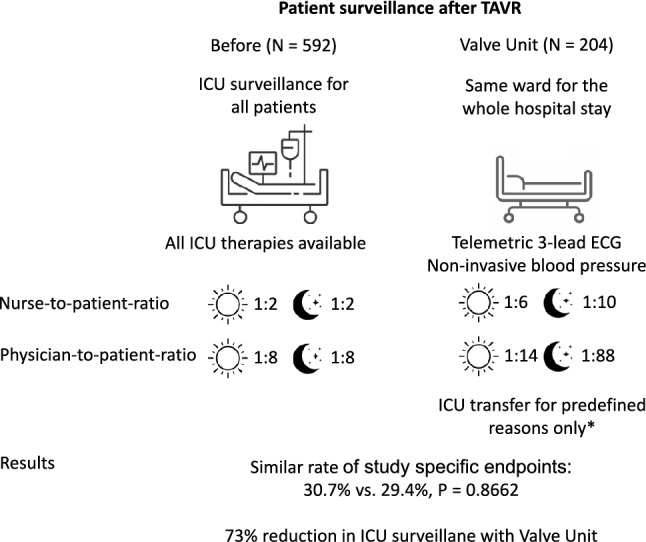

## Introduction

Due to the current lack of guidelines regarding the postprocedural surveillance of patients after TAVR, relevant discrepancies between institutions exist. The standard of care at most institutions in Germany and around the globe is routine surveillance at an intensive care unit (ICU) or intermediate care unit for 24–48 h. TAVR is now a common procedure in clinical practice and complication rates have decreased significantly during its over 20-year history. This led some centers to recommend transferring patients at low risk for complications to the normal wards immediately after implantation of the new valve [1].

To evaluate if postprocedural monitoring at a valve unit (VU) instead of routine ICU monitoring (ICM) would change the rate of adverse events we planned a prospective case–control study to evaluate adverse event rates of patients directly before and after implementation of a change in the postprocedural care pathway at our institution. With the new postprocedural care pathway patients who did not meet pre-specified criteria for ICU monitoring were transferred to a newly established VU integrated into our cardiology wards. Our aim was to evaluate if surveilling patients at a VU after TAVR would reduce the necessity for intensive care monitoring without increasing the rate of adverse events.

## Methods

### Study design

In this prospective case–control study, 796 consecutive patients treated with transfemoral TAVR for aortic valve disease between January 2019 and December 2020 were included. The decision for transfemoral TAVR was made by the interdisciplinary heart team according to the 2017 European Society of Cardiology/European Association for Cardio-Thoracic Surgery Guidelines for the management of valvular heart disease [2]. The study was approved by the local ethics committee. All patients gave written informed consent.

### The valve unit

Prior to the implementation of a VU for post-interventional monitoring, all patients at our institution received routine post-interventional monitoring at our intensive care unit (ICU) for at least 24 h after TAVR, as was routine in Germany.

We established a dedicated VU in April 2020 for the pre- and post-interventional care of our TAVR patients. A goal was for patients to stay in the same hospital room and be treated by the same medical team throughout their procedure. The VU was spatially integrated into a general cardiology ward at our tertiary care center and equipped with continuous telemetric ECG, periodical non-invasive blood pressure monitoring, and peripheral oxygen saturation connected to a central alarm system. All patients who did not meet predefined criteria for intensive care surveillance after the procedure were immediately transferred from the hybrid catheter laboratory back to the VU after vascular closure was achieved. Periprocedural management at the VU was standardized with predefined timepoints and standards for laboratory tests, informed patient consent, CT imaging, pre-interventional assessment by an anesthesiologist, pausing of oral anticoagulation, pre- and post-interventional echocardiography, as well as early mobilization.

The nurse-to-patient ratio at the VU was 1:6 during the day shift and 1:10 during the night shift. The physician-to-patient ratio was 1:14 during the hours of 8 a.m. to 5 p.m. and 1:88 after hours, with a single resident overseeing all patients in the cardiology wards.

Indications for ICU transfer were hemodynamic instability (systolic blood pressure below 80 mmHg, the requirement for catecholamine treatment, or the necessity for left ventricular assist device implantation), respiratory instability (Ohio mask with more than 6 L of oxygen per minute or the need for invasive or non-invasive ventilation), severe rhythm disorders (e.g. new third-degree atrioventricular block), periprocedural stroke, delirium, severe bleeding complications, periprocedural myocardial infarction. Of the 796 patients included in the study, 592 had been treated before the change in the postprocedural care pathway and 204 afterwards.

### TAVR and clinical parameters

TAVR was performed under conscious sedation in a hybrid catheterization laboratory by a team of two experienced operators, two catheter nurses and an anesthesia team using a standardized procedure protocol.

The baseline characteristics of each patient, including their medications and medical history, were recorded along with the clinically relevant periprocedural information. To screen for postinterventional atrioventricular block, all patients received daily 12-lead ECGs in addition to telemetric monitoring. Repeated laboratory testing and clinical examinations were performed to screen for renal failure, infection as well as signs of bleeding. Before being discharged, all patients underwent transthoracic echocardiography to measure the transvalvular aortic valve gradient as well as evaluate paravalvular aortic regurgitation.

### Statistical analysis

Statistical analysis was performed using MedCalc software (MedCalc Version 20.210, MedCalc Software Ltd, Ostend; Belgium). Continuous variables are expressed as mean ± standard error of the mean and were compared by t-test. Categorical variables are presented as counts and percentages and differences between proportions were calculated by using the *χ*^2^ test. A logistic univariate and multivariate regression analysis was performed to identify predictors for in-hospital complications after TAVR, and results are presented as an odds ratio with a 95% confidence interval (CI). All statistical tests were two-sided and *P*-value below 0.05 was considered statistically significant.

## Results

### Study cohort and rate of ICU surveillance

**Fig. 1:**
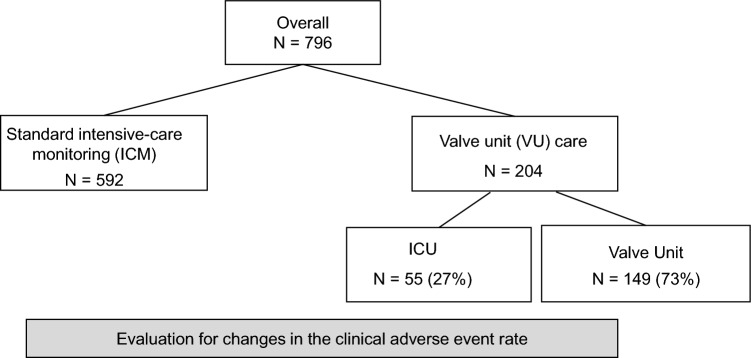
Flow chart During standard intensive care monitoring all patients received ICU surveillance. Starting in April 2020, patients without predefined criteria for ICU surveillance were transferred back to a valve unit immediately after TAVR. We evaluated if switching from ICM to VU care changed the frequency of clinical adverse events

Of the 796 patients included in the study 592 were treated during ICM; all of whom (100%) received surveillance at our ICU after aortic valve implantation (see Fig. 1 for a flow chart). During VU monitoring, 204 patients were treated with TAVR. 55 of them (27%) fulfilled the criteria for ICU transfer and were transferred accordingly after the procedure. 149 (73%) did not meet ICU criteria and were transferred to the VU. Reasons for ICU transfer after establishment of the VU care were 3rd degree atrioventricular block (*n* = 27), hemodynamic instability (*n* = 20), vascular complications (*n* = 3), neurologic complications (*n* = 4), and acute coronary syndrome (*n* = 1).

VU patients transferred to the ICU did not have a significantly higher STS-PROM compared to the majority of patients who did not need ICU surveillance. The STS-PROM of VU patients with ICU transfer was 4.45 ± 3.62 compared to 3.47 ± 2.99 for VU patients without ICU transfer (*P* = 0.226).

### Baseline patient characteristics

There were no significant differences between the two groups in age, sex, rate of diabetes mellitus, body mass index, history of myocardial infarction, atrial fibrillation, percentage of patients with New York Heart Association (NYHA) functional status III and IV, Society of Thoracic Surgeons risk score (STS-score) for postoperative mortality, type of implanted TAVR prosthesis, left ventricular ejection fraction, aortic valve area or aortic annulus area (see Table 1).

**Table 1:** Patient characteristics during ICM and VU

	Standard intensive-care monitoring (ICM)	Valve unit (VU)	*P* value
Number of patients	592	204	
Age, years	80.0 ± 6.3	79.2 ± 11.6	0.28
Female	277 (46.8%)	100 (49.0%)	0.67
Diabetes mellitus	162 (27.4%)	51 (25%)	0.43
BMI, kg/m^2^	27.5 ± 5.2	26.6 ± 3.8	0.19
History of myocardial infarction	57 (9.6%)	15 (7.4%)	0.54
Atrial fibrillation	210 (35.5%)	68 (33.3%)	0.61
Chronic renal failure	120 (20.3%)	56 (27.5%)	***0.03***
NYHA class III/IV	402 (67.9%)	131 (64.2%)	0.33
STS-PROM	3.5 ± 2.5	3.7 ± 3.2	0.46
Implanted TAVR prosthesis			
Medtronic Evolut R/PRO	228 (38.5%)	74 (36.3%)	0.61
Edwards Sapien S3	171 (28.9%)	52 (25.5%)	0.37
Lotus Edge	185 (31.3%)	78 (38.2%)	0.07
Acurate Neo	8 (1.4)	0 (0%)	0.21
Baseline echocardiographic criteria			
Ejection fraction, %	51.3 ± 19.3	51.5 ± 10.5	0.92
Aortic valve area, cm^2^	0.8 ± 0.2	0.78 ± 0.2	0.38
AV mean PG, mmHg	39.4 ± 14.5	42.8 ± 14.9	***0.007***
Aortic anulus area, cm^2^	24.4 ± 2.4	23.8 ± 2.1	0.79

*BMI* body mass index, *NYHA* New York Heart Association, *STS-PROM* Society of Thoracic Surgeons predicted risk of mortality, *AV* mean *PG* aortic valve mean pressure gradient

*P*-value < 0.05 was deemed statistically significant and highlighted in bolditalic

### Study-specific endpoints

The overall rate of study-specific endpoints was similar before and after the implementation of the VU (Table 2). During ICM, 182 of 592 patients developed study-specific endpoints (30.7%) compared to 60 of 204 patients (29.4%) after the introduction of the VU (*P* value for difference: 0.82). The most common endpoints were need for permanent pacemaker implantation (ICM 14.7% vs VU 15.2%, *P* value 0.86), pneumonia or other infection treated with intravenous antibiotics (ICM 4.9% vs VU 4.9%, *P* value 1.0) and stroke or transient ischemic attacks (ICM 2.9% vs VU 3.4%, *P* value 0.69). When patients were not transferred to the ICU routinely, a trend for a lower rate of delirium (ICM 3.5% vs VU 1%, *p*-value 0.06) was observed.

**Table 2:** Summary of study-specific endpoints during standard intensive-care (ICM) and valve unit (VU) care

Complications	Standard intensive-care monitoring (*N* = 592)	Valve unit (*N* = 204)	*P* value
Permanent pacemaker	87 (14.7%)	31 (15.2%)	0.8626
Pneumonia or other infection	29 (4.9%)	10 (4.9%)	0.9985
Stroke or TIA	17 (2.9%)	7 (3.4%)	0.6873
Delirium	21 (3.5%)	2 (1%)	0.0592
Acute kidney injury	3 (0.5%)	5 (2.5%)	***0.0163***
Bleeding, major	3 (0.5%)	1 (0.5%)	0.9770
Vascular complications, major	1 (0.2%)	0 (0%)	0.5572
Myocardial infarction	1 (0.2%)	0 (0%)	0.5572
Acute heart failure	9 (1.5%)	1 (0.5%)	0.2552
CPR	5 (0.8%)	0 (0%)	0.1882
Death	5 (0.8%)	3 (1.5%)	0.4311
Other	1 (0.2)	0 (0%)	0.5572
Overall	182 (30.7%)	60 (29.4%)	0.8662

*CPR* cardiopulmonary resuscitation, *TIA* transient ischemic attacks

*P*-value < 0.05 was deemed statistically significant and highlighted in bolditalic

### Predictors of in-hospital complications

To test for predictors of complications, univariate logistic regression was performed. In-hospital complications following TAVR were found to be associated with chronic pulmonary disease, NYHA class, NTproBNP levels and pre-existing anemia (Table 3). In the absence of guideline recommendations, patients with severe pre-existing anemia (hemoglobin < 8 g/dl) at our institution received blood transfusion with a hemoglobin target of > 9 g/dl, and iron deficiency was treated with supplementation to account for potential blood loss during the procedure. With multivariate logistic regression only pre-existing chronic pulmonary disease still met statistical significance (odds ratio 1.66 (95% CI 1.17–2.37), *P* Value 0.01).

**Table 3:** Predictors of in-hospital complications in the overall patient population

Logistic regression	Univariate		Multivariate	
	OR (95% CI)	*p*-value	OR (95% CI)	*P* value
Age	1.02 (0.99–1.04)	0.12		0.09
Female	0.98 (0.73–1.33)	0.69	–	–
BMI	1.00 (0.97–1.04)	0.9	–	–
Diabetes mellitus	1.14 (0.91–1.59)	0.46		0.98
Chronic pulmonary disease	1.52 (1.11–2.08)	***0.009***	1.66 (1.17–2.37)	***0.01***
Atrial fibrillation	1.14 (0.83–1.56)	0.41		0.76
NYHA class	1.22 (1.0–1.48)	***0.047***		0.15
History of stroke/TIA	1.33 (0.85–2.07)	0.22		0.14
NTproBNP	per pg/ml	0.02		0.27
GFR	per ml/min	0.59	–	–
Pre-existing anemia	1.64 (1.02–2.65)	***0.04***		0.21

*OR* odds ratio, *95% CI* 95% confidence interval, *BMI* body mass index, *NYHA* New York Heart Association, TIA transient ischemic attack, *GFR* glomerular filtration rate

*P*-value < 0.05 was deemed statistically significant and highlighted in bolditalic

## Discussion

The main findings of this study are: 1. Monitoring at the VU did not increase adverse event rates after TAVR compared to ICM. 2. Introduction of the VU reduced the percentage of postinterventional ICU admissions by 73%. 3. A trend towards a lower rate of delirium was observed for patients in the VU group.

During the two decades since Cribier et al. reported the first successful TAVR performed in a patient in 2002, procedural planning, TAVR devices, methods for vascular closure and clinical experience have dramatically improved. This led to an expansion of TAVR indications and a substantial increase in TAVR procedures worldwide over time. This increased the need for a simplified and optimized TAVR approach without compromising safety and efficacy to treat patients at a reasonable economic cost [3]. Optimization of the discharge management [4] to reduce the length of the postinterventional hospital stay as well as omitting ICU transfer [5] in low-risk patients are major ways of achieving that goal. The 3M [6] and the recently published Optimize PRO study [7] demonstrated that standardized periprocedural protocols can reduce rates of major complications.

Intensive care beds are a limited resource and, therefore, should only be utilized by patients with a need for increased surveillance. Whenever possible and safe patients should, therefore, be transferred to less resource-intensive wards. TAVR has become a routine procedure and the chances of serious complications have significantly declined from the early days. However, complications like 3rd degree AV-block necessitating permanent pacemaker implantation are still common. We assumed that most patients could be safely monitored at a VU integrated into our general cardiology wards with 24-h telemetric rhythm monitoring capabilities in addition to non-invasive blood pressure monitoring. Patients with periinterventional complications like 3rd degree AV-block or cardiovascular instability were still admitted to the ICU for postinterventional surveillance until stabilization.

Introduction of the VU reduced the percentage of postinterventional ICU admissions by 73%. There were no significant differences in major complications between patients in the ICM and VU group. A direct comparison of major complications of patients having received routine ICU surveillance compared to patients treated at the newly established VU did not show significant between-group differences except for the rate of acute renal failure. The most likely reason for the increased rate of acute renal failure in the VU group is the 7.3% higher baseline rate of chronic renal failure. A numerically lower rate of post-interventional delirium favoured care at the VU.

The implementation of standardized techniques aimed at minimizing periprocedural complications, such as sonographic or angiographic vascular puncture and the evaluation of successful closure, alongside a uniform approach to periprocedural surveillance and diagnostics, has been demonstrated to decrease hospital length of stay without reducing safety [4, 8–11]. A dedicated VU enables the efficient implementation and continuous improvement of quality measures. The introduction of the VU at our institution further optimized these factors. This study did not evaluate the impact of introducing a VU on the length of hospital stays. The implementation of the VU at our institution coincided with the SARS-CoV-2 pandemic, resulting in significant delays in diagnostics, scheduling of non-emergent procedures, and patient discharges. Further investigation is necessary to determine if VU can reduce the length of hospital stays. The country in which a hospital is situated, along with its healthcare system and payment structure, significantly impacts the duration of hospital stays and the decision regarding whether a patient is discharged home or transferred to another facility for continued care, even when similar strategies to minimize length of stay are implemented [4].

Our study should be considered hypothesis-generating, since future adequately powered multicenter studies are required to confirm that postinterventional surveillance after TAVR at a VU is safe and to assess the optimal setup of a VU.

In summary, transferring patients without risk factors directly after TAVR to a VU equipped with 24-h ECG and non-invasive blood pressure monitoring instead of ICU surveillance appears to be safe and a further step forward toward fast-track TAVR.

## Data Availability

The data that support the findings of this study are available from the corresponding author, D.B., upon reasonable request.

## Abbreviations

VU: Valve unit
ICM: Intensive care monitoring
ICU: Intensive care unit
TAVR: Transcatheter aortic valve replacement
ECG: Electrocardiogram
STS-score: Society of Thoracic Surgeons risk score
NYHA: New York Heart Association
n: Number of patients
